# Supplementary material for: Epidemiology of the SARS-CoV-2 Omicron Variant Emergence in the Southeast Brazilian Population
Source: Microorganisms. 2024 Feb 23;12(3):449. doi: 10.3390/microorganisms12030449 (PMC10974166; doi:10.3390/microorganisms12030449)
Supplement: Supplementary file 1 [file microorganisms-12-00449-s001.zip › Supplementary Table S1.pdf]

Supplementary Table S1. Epidemiological characteristics of SARS-CoV-2 evaluated individuals.

	Epidemiological week of 2022										
	**1st** 02-08 Jan.	**2nd** 09-15 Jan.	**3rd** 16-22 Jan.	**4th** 23-29 Jan.	**5th** 30 Jan. to 05 Feb.	**6th** 06-12 Feb.	**7th** 13-19 Feb.	**8th** 20-26 Feb.	**9th** 27 Feb. to 05 Mar.	**10th** 06-12 Mar.	**Total**
**N° SARS-CoV-2 Molecular tests**	5,398	6,618	6,606	5,567	4,224	3,824	4,020	3,444	1,855	1,650	43,206
**N° of positive SARS-CoV-2 (%)**	1290 (23.9)	2604 (39.3)	3093 (46.8)	3321 (59.7)	2479 (58.7)	1865 (48.8)	1554 (38.7)	916 (26.6)	361 (19.5)	212 (12.8)	17,695 (40.9)
**Mean age (SD) Positive SARS-CoV-2**	35.4 (16.2)	36.1 (16.2)	38.3 (16.7)	38.3 (17.3)	38.3 (17.7)	37.1 (17.7)	35.7 (18.7)	35.7 (19.5)	37.5 (19.6)	37.0 (19.1)	-
**0-10y N°(%)**	37 (2.9)	55 (2.1)	83 (2.7)	134 (4.0)	123 (5.0)	95 (5.1)	130 (8.4)	84 (9.2)	23 (6.4)	15 (7.1)	779 (4.4)
**11-19y N°(%)**	127 (9.8)	240 (9.2)	235 (7.6)	243 (7.3)	214 (8.6)	188 (10.1)	180 (11.6)	136 (14.8)	46 (12.7)	24 (11.3)	1,633 (9.2)
**20-59y N°(%)**	1007 (78.1)	2060 (79.1)	2429 (78.5)	2525 (76)	1826 (73.7)	1369 (73.4)	1059 (68.1)	588 (64.2)	235 (65.1)	146 (68.9)	13,244 (74.8)
**>60y N°(%)**	119 (9.2)	249 (9.6)	346 (11.2)	419 (12.6)	316 (12.7)	213 (11.4)	185 (11.9)	108 (11.8)	57 (15.8)	27 (12.7)	2,039 (11.5)

**Gender**	*Male (%)*	500 (38.8)	1154 (44.3)	1302 (42.1)	1464 (44.1)	1083 (43.7)	803 (43.0)	639 (41.1)	364 (39.8)	140 (38.8)	86 (40.6)	7,535 (42.6)
	Female (%)	790 (61.2)	1450 (55.7)	1791 (57.9)	1857 (55.9)	1396 (56.3)	1062 (57.0)	915 (58.9)	552 (60.2)	221 (61.2)	126 (59.4)	10,160 (57.4)

N° =number of samples; N° SARS-CoV-2 molecular tests =number of samples tested with SARS-CoV-2 molecular diagnosis; Gender = gender of positive SARS-CoV-2.
