# Supplementary figures and images for: Epidemiology of the SARS-CoV-2 Omicron Variant Emergence in the Southeast Brazilian Population
Source: Microorganisms. 2024 Feb 23;12(3):449. doi: 10.3390/microorganisms12030449 (PMC10974166; doi:10.3390/microorganisms12030449)

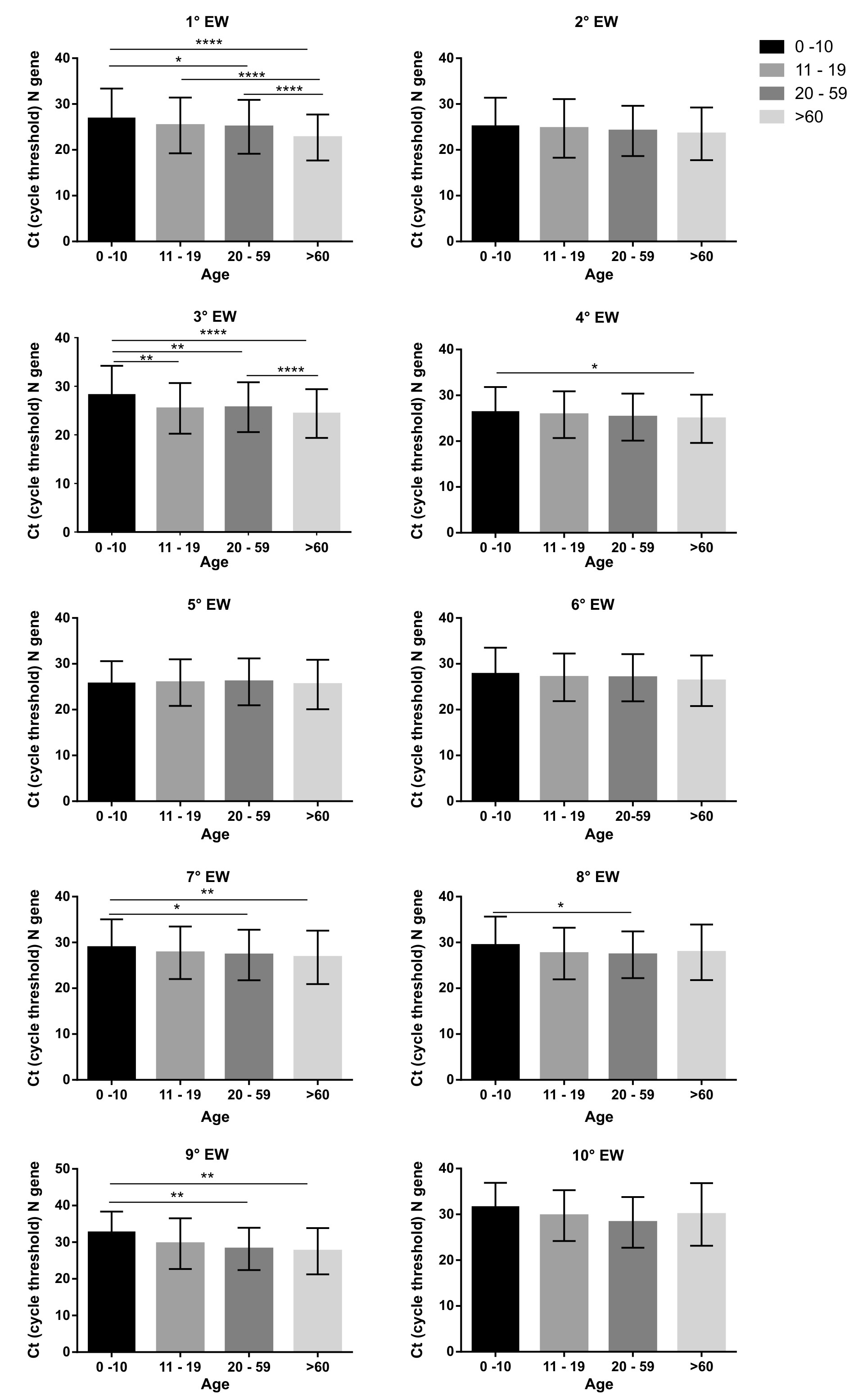

Supplement: Supplementary file 1 [file microorganisms-12-00449-s001.zip › Supplementary Figure S1.jpg]
